# SNP Discovery for mapping alien introgressions in wheat

**DOI:** 10.1186/1471-2164-15-273

**Published:** 2014-04-10

**Authors:** Vijay K Tiwari, Shichen Wang, Sunish Sehgal, Jan Vrána, Bernd Friebe, Marie Kubaláková, Praveen Chhuneja, Jaroslav Doležel, Eduard Akhunov, Bhanu Kalia, Jamal Sabir, Bikram S Gill

**Affiliations:** 1 Wheat Genetics Resource Center, Department of Plant Pathology, Kansas State University, Manhattan, KS 66506, USA; 2 Department of Plant Pathology, Kansas State University, Manhattan, KS 66506, USA; 3 Institute of Experimental Botany, Centre of the Region Haná for Biotechnological and Agricultural Research, Olomouc CZ-78371, Czech Republic; 4 School of Agricultural Biotechnology, Punjab Agricultural University, Ludhiana, India; 5 Department of Biological Sciences, Faculty of Sciences, King Abdulaziz University, Zeddah, Saudi Arabia

## Abstract

**Background:**

Monitoring alien introgressions in crop plants is difficult due to the lack of genetic and molecular mapping information on the wild crop relatives. The tertiary gene pool of wheat is a very important source of genetic variability for wheat improvement against biotic and abiotic stresses. By exploring the 5M^g^ short arm (5M^g^S) of *Aegilops geniculata,* we can apply chromosome genomics for the discovery of SNP markers and their use for monitoring alien introgressions in wheat (*Triticum aestivum* L).

**Results:**

The short arm of chromosome 5M^g^ of *Ae. geniculata* Roth (syn. *Ae. ovata* L.; 2n = 4x = 28, U^g^U^g^M^g^M^g^) was flow-sorted from a wheat line in which it is maintained as a telocentric chromosome. DNA of the sorted arm was amplified and sequenced using an Illumina Hiseq 2000 with ~45x coverage. The sequence data was used for SNP discovery against wheat homoeologous group-5 assemblies. A total of 2,178 unique, 5M^g^S-specific SNPs were discovered. Randomly selected samples of 59 5M^g^S-specific SNPs were tested (44 by KASPar assay and 15 by Sanger sequencing) and 84% were validated. Of the selected SNPs, 97% mapped to a chromosome 5M^g^ addition to wheat (the source of t5M^g^S), and 94% to 5M^g^ introgressed from a different accession of *Ae. geniculata* substituting for chromosome 5D of wheat. The validated SNPs also identified chromosome segments of 5M^g^S origin in a set of T5D-5M^g^ translocation lines; eight SNPs (25%) mapped to TA5601 [T5DL · 5DS-5M^g^S(0.75)] and three (8%) to TA5602 [T5DL · 5DS-5M^g^S (0.95)]. SNPs (*gsnp_5ms83* and *gsnp_5ms94*), tagging chromosome T5DL · 5DS-5M^g^S(0.95) with the smallest introgression carrying resistance to leaf rust (*Lr57*) and stripe rust (*Yr40*), were validated in two released germplasm lines with *Lr57* and *Yr40* genes.

**Conclusion:**

This approach should be widely applicable for the identification of species/genome-specific SNPs. The development of a large number of SNP markers will facilitate the precise introgression and monitoring of alien segments in crop breeding programs and further enable mapping and cloning novel genes from the wild relatives of crop plants.

## Background

Crop plants have a narrow genetic base because of domestication and breeding. Moreover, many crop plants have gone through a polyploidization bottleneck. Introgressive hybridization with wild relatives, often described as alien introgression, is widely used to broaden the crop genetic base. Bread or hexaploid wheat (*Triticum aestivum* L., 2n = 6x = 42, AABBDD genome), which accounts for 95% of the harvested wheat crop, traces its origin to a rare hybridization event ~6,000 years ago involving *T. turgidum* L. (2n = 4x = 28, AABB) and *Aegilops tauschii* Coss. (2n = 2x = 14, DD) [1-3]. McFadden and Sears [1], Kihara [2], and McFadden and Sears [3] have reproduced this hybridization event to generate ‘synthetic wheat’. These two species, and wheat landraces, constitute the primary gene pool of wheat [4]. Synthetic wheats and direct crosses between *T. aestivum* and *Ae. tauschii *[5,6] have been used to enrich the bread wheat genetic base. Extensive wheat genetic resources and marker systems [7-9] are transferable and can be used for mapping of alien introgressions [10], characterizing genetic diversity [11] and gene isolation [12,13] from the primary gene pool.

Tetraploid, emmer wheat *T. turgidum* L. arose ~350,000 years ago from a hybridization between *T. urartu* Tuanian ex Gandilyan (2n = 2x = 14, AA) and a B-genome species, whose closest living relative is *Ae. speltoides* Tausch (2n = 2x = SS) [14-16]. These two species, together with the A-genome species *T. monococcum* L. subsps. *monococcum* and *aegilopoides*, the tetraploid wheat sibling species *T. timopheevii* Zhuk. (2n = 4x = 28, AAGG) and D genome cluster present in polyploid *Aegilops* species [17] constitute the secondary gene pool. Usually, wheat marker systems can be used to map alien introgressions from secondary gene pool.

Hundreds of other species in the *Triticeae* tribe contain genomes other than A, B and D, and these species constitute the tertiary gene pool of bread wheat. All of these species can be hybridized with hexaploid wheat to produce amphiploids, addition and translocation lines [18-20]. Chromosome engineering approaches have been used [4,21-24] to produce small alien transfers without linkage drag. Cytological approaches have been extensively used to identify alien introgression lines. However, these approaches lack throughput and resolution and are not suitable when analyzing a very large number of progeny for detecting a rare recombination event [25].

Molecular markers can detect small chromosome segments not detectable cytologically and permit easier identification of the introgressed alien fragments. Microsatellite markers have been used extensively in the primary and secondary gene pools, however, they have low transferability to tertiary gene pool species, and the lack of locus specificity hampers their application [26]. Wheat EST bin maps have been explored as a source of markers, but polymorphic markers are rare [4]. Single-nucleotide polymorphism (SNP) markers have become the technology of choice for all organisms because of their wide distribution in genomes and compatibility with high, multiplex detection systems [27-31]. Advances in SNP marker development in wheat and the availability of various SNP genotyping platforms now permit high-throughput and cost-effective genotyping [27,28,31].

Despite the progress in DNA marker technology, mapping large and polyploid genomes such as wheat remains a daunting task. Mapping and sequencing complex plant genomes can be simplified by dissecting the chromosomes by flow cytometric sorting [32]. This approach reduces sample complexity and enables analysis at the subgenomic level. Flow cytometric chromosome sorting has been implemented successfully in many plant species, including cultivated cereals (such as bread and durum wheat), barley, rye, oats, rice and maize [33]. Recently, Molnár et al. reported flow-sorting of individual chromosomes from *Ae. umbellulata* Zhuk. (2n = 2x = 14, UU) and *Ae. comosa* Sm. In Sibth. & Sm. (2n = 2x = 14, MM) and from their natural allotetraploid hybrids (*Ae. biuncialis* Vis. and *Ae. geniculata*) [34,35]*.* This study provided opportunity for the next-generation sequencing of individual *Aegilops* chromosomes for the development of sequence-based markers and their application in wheat breeding.

*Ae. geniculata*, also called ovate goatgrass, is found widely distributed in the Middle East. A member of the tertiary gene pool of wheat, *Ae. geniculata* arose from hybridization between the diploid species *Ae. umbellulata* and *Ae. comosa *[36,37] and is an important source of useful genes for wheat improvement [20,38-41]. The *Ae. geniculata* genome has been introgressed into wheat, and single-chromosome, addition lines were developed by Friebe et al. [42]. Previously, we have reported on the transfer of genes *Lr57 and Yr40 *[43] and *Sr53 *[44] from chromosome 5M^g^ of *Ae. geniculata* to chromosome 5D of wheat. In this study, we present the first report on alien chromosome-based SNP discovery and its application in mapping of alien introgression in wheat.

## Results

### Flow-sorting and sequencing of the 5M^g^ short arm

**Figure 1:**
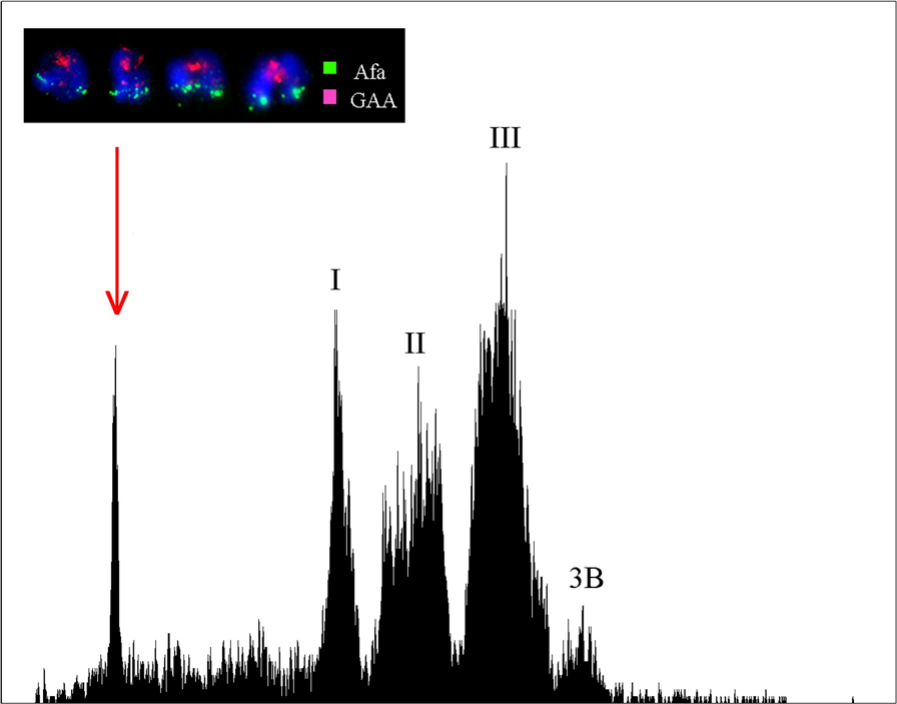
**Flow cytometric analysis of 5M**^**g **^**short arm.** Histogram of the relative fluorescence intensity (flow karyotype) obtained after the analysis of a DAPI-stained chromosome suspension prepared from a wheat-*Ae. geniculata* ditelosomic addition line t5M^g^S. The leftmost peak represents chromosome t5M^g^S. In addition, the flow karyotype comprises three composite peaks I, II and III, representing groups of wheat chromosomes, and a peak representing wheat chromosome 3B. Inset: Examples of flow sorted chromosomes after FISH with probes for the Afa-family (yellow-green) and [GAA]n repeats (red). The chromosomes were counterstained by DAPI (blue). X axis: DAPI fluorescence intensity; Y axis: number of events.

The analysis of DAPI-stained, chromosome suspensions prepared from a wheat–*Ae. geniculata* t5M^g^S telocentric addition line resulted in histograms with five peaks of fluorescence intensity (flow karyotypes) (Figure 1). The leftmost peak represents telochromosome t5M^g^S, which was well resolved from composite peaks I, II, III and peak 3B of the bread wheat chromosomes; t5M^g^S was flow-sorted with 92.6% purity. A random mix of chromosome and chromatid fragments contaminated the sorted fractions. DNA amplified from flow-sorted t5M^g^S was sequenced by the Illumina technology. In total, we generated more than 153 million reads of 100 bp from one-lane HiSeq sequencing. After quality trimming and filtering, about 145 million reads (~95%) were used for mapping, providing approximately ~45x coverage for t5M^g^S. *De novo* assembly of the 5M^g^S reads resulted in 7,319 contigs with length ≥ 500 bp and average coverage depth of 20x (Additional file 1: Figure S1). Blast against wheat EST sequences showed that 1,408 of the 7,319 contigs may contain genes. Sequence data generated in this study was submitted to SRA database (accession: SRX474187).

### Mapping of reads and SNP calling

For mapping the 5M^g^S reads, we used 5AS, 5BS and 5DS chromosome shotgun sequence assemblies provided by the International Wheat Genome Sequencing Consortium. A maximum of three mismatches were allowed for each read for mapping (Additional file 2: Figure S2) on reference group of chromosome five short arm assemblies (5AS, 5BS and 5DS). Depending on the references used to map the reads, only about 30%, 23% and 25% of reads could be mapped to the 5AS, 5BS and 5DS assemblies, respectively. The mapped 5M^g^S reads covered 103,203,161 (52%) of the 5AS, 93,086,474 (53%) of the 5BS and 69,678,915 (47%) of the 5DS assemblies (Additional file 2: Figure S2). Based on the alignments of the 5M^g^S reads, we discovered 976,754 5AS, 675,007 5BS and 851,722 5DS raw variations. After filtering with a coverage depth of 4 and a SNP quality of 50; 277770 (5AS), 203522 (5BS) and 355765 (5DS) high-quality SNPs were retained, which were used for further analysis after analyzing 220624667 base pair sequences in total (Table 1). SNP densities of the 5M^g^S sequences against 5AS, 5BS and 5DS were observed to be 1.3, 0.9 and 1.6 SNPs/kb, respectively, with an average of 1.3 SNP/kb. We searched against the wheat EST and NCBI nr databases with blastn and blastx and discovered 35749 (5AS), 31526 (5BS) and 11704 (5DS) SNPs located in the gene coding regions.

**Table 1:** **SNPs derived by comparing 5M**^**g**^**S sequences based on reads mapped on the 5AS, 5BS and 5DS reference sequence assemblies from the IWGSC**

**Reference assemblies**	**Mapped reads**	**Raw SNPs**	**Filtered SNPs**	**Genic SNPs**
**5AS**	45,989,152	976,754	277,770	35,749
**5BS**	35,168,202	675,007	203,522	31,526
**5DS**	38,012,773	851,722	355,765	11,704

### 5M^g^-genome-specific SNPs

After mapping the 5M^g^S reads from *Ae. geniculata* on the group-5, short-arm assemblies of Chinese Spring, we focused on finding 5M^g^-specific SNPs. SNPs that had the same alleles on 5A, 5B and 5D but a different allele on 5M^g^ were identified. It was found that 2,178 SNPs have the same alleles in the 5AS, 5BS and 5DS contigs and different alleles in the 5M^g^S sequences. These SNPs were putative 5M^g^-specific SNPs (for convenience, we only kept the SNP positions belonging to 5AS contigs). To make sure that the flanking sequences of 5M^g^S-specific SNPs matched the 5M^g^S assemblies, flanking sequences of 5M^g^S-specific SNPs were blasted to the 5M^g^S assemblies so that the primers designed based on the flanking sequences would work for both 5A (5B, 5D) and 5M^g^. To eliminate interference from variations that may locate in the flanking sequences, only 104 SNPs that showed no variation in the 100-bp flanking sequences of SNPs between the 5A and 5M^g^S contigs were selected (Additional file 3: Table S1).

### SNP validation

**Figure 2:**
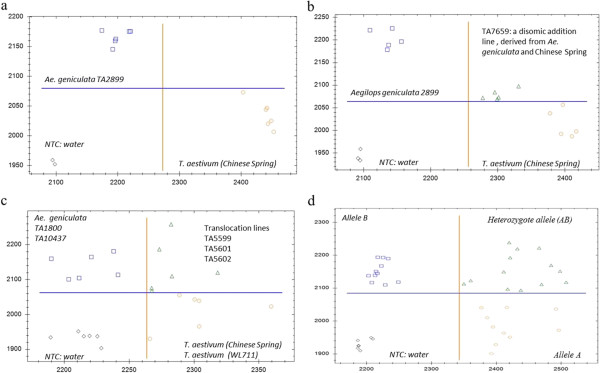
**(a-d) Test of KASPar markers on different plant materials. a**. Test of markers on *Ae. geniculata* TA2899 and Chinese Spring. **b**. A KASPar assay on set of parental lines and the disomic addition line of 5 M from *Ae. geniculata* TA2899. **c**. Test of a KASPar marker on a set of translocation lines derived from *Ae. geniculata* TA10437 and *T. aestivum* cv. WL711. **d**. Test of a polymorphic KASPar marker between *Ae. geniculata* TA1800 and TA10437 and an F2 population (32 lines tested) derived from crossing them.

In order to analyze the authenticity of the discovered SNPs, 44 sequences with one SNP each were randomly chosen from SNP sequences and used to design a KASPar genotyping assay. Of the 44 KASPar genotyping assays, six SNPs had identical alleles in wheat and *Ae. geniculata* and two SNPs showed heterozygous alleles in *Ae. geniculata* accession TA2899. On average, 84% SNPs were validated in Chinese Spring and the *Ae. geniculata* TA2899 (Figure 2a).

For Sanger sequencing-based validation, we tested 15 primers derived from SNP sequences; three did not show any amplification in either line suggesting the need for PCR optimization. Of 12 primers tested between Chinese Spring and *Ae. geniculata* TA2899, the SNP validation rate was ~84%, which was similar to the results from the KASPar assay.

KASPar assays were done on wheat cultivars Chinese Spring, WL711 and seven *Ae. geniculata* accessions to test the applicability of the 44 candidate SNPs (Table 2). Sixty-nine to 85% of the SNPs showed different alleles between wheat and the *Ae. geniculata* lines (Table 2). Approximately 10% of the SNPs were polymorphic between *Ae. geniculata* accessions TA1800 and TA10437, and polymorphic SNPs were tested on a subset of an F_2_ population (36 lines) developed from a cross between TA1800 and TA10437 (Figure 2d). Of a total of 59 SNPs tested, 33 belonged to genic sequences and 26 belonged to non-genic sequences. SNP validation rates were similar for both genic (86%) and non-genic sequences.

**Table 2:** **Validation percentage of 44 5M**^**g**^**S-specific SNPs on a set of seven *Ae. geniculata *lines against two wheat cultivars (*T. aestivum cv. *Chinese Spring and WL711)**

**Wheat cultivars**	**Validation of 5M**^**g**^**S-specific SNPs in selected *Ae. geniculata *accessions (%)**						
	**TA2899**	**TA10437**	**TA1800**	**TA1801**	**TA2786**	**TA2041**	**TA10029**
**Chinese spring**	85.4	83.7	83.3	78.4	78.8	69.4	68.8
**WL711**	75.0	81.4	81.4	78.8	76.5	73.0	65.7

### Application of 5M^g^-specific SNPs

#### Addition lines

Validated SNPs between Chinese Spring and *Ae. geniculata* accession TA2899 were used to identify 5M^g^ chromosome in the chromosome complement of Chinese Spring wheat. In the 5M^g^ addition line, 5M^g^-specific SNPs were expected to have heterozygous condition, because wheat chromosomes 5A, 5B and 5D carry the alternative allele. Out of 37 SNPs, 97% detected heterozygous alleles (Figure 2b), confirming the presence of both wheat and *Ae. geniculata* chromosomes in the tested addition line (TA7659).

#### *Substitution and translocation lines*

**Figure 3:**
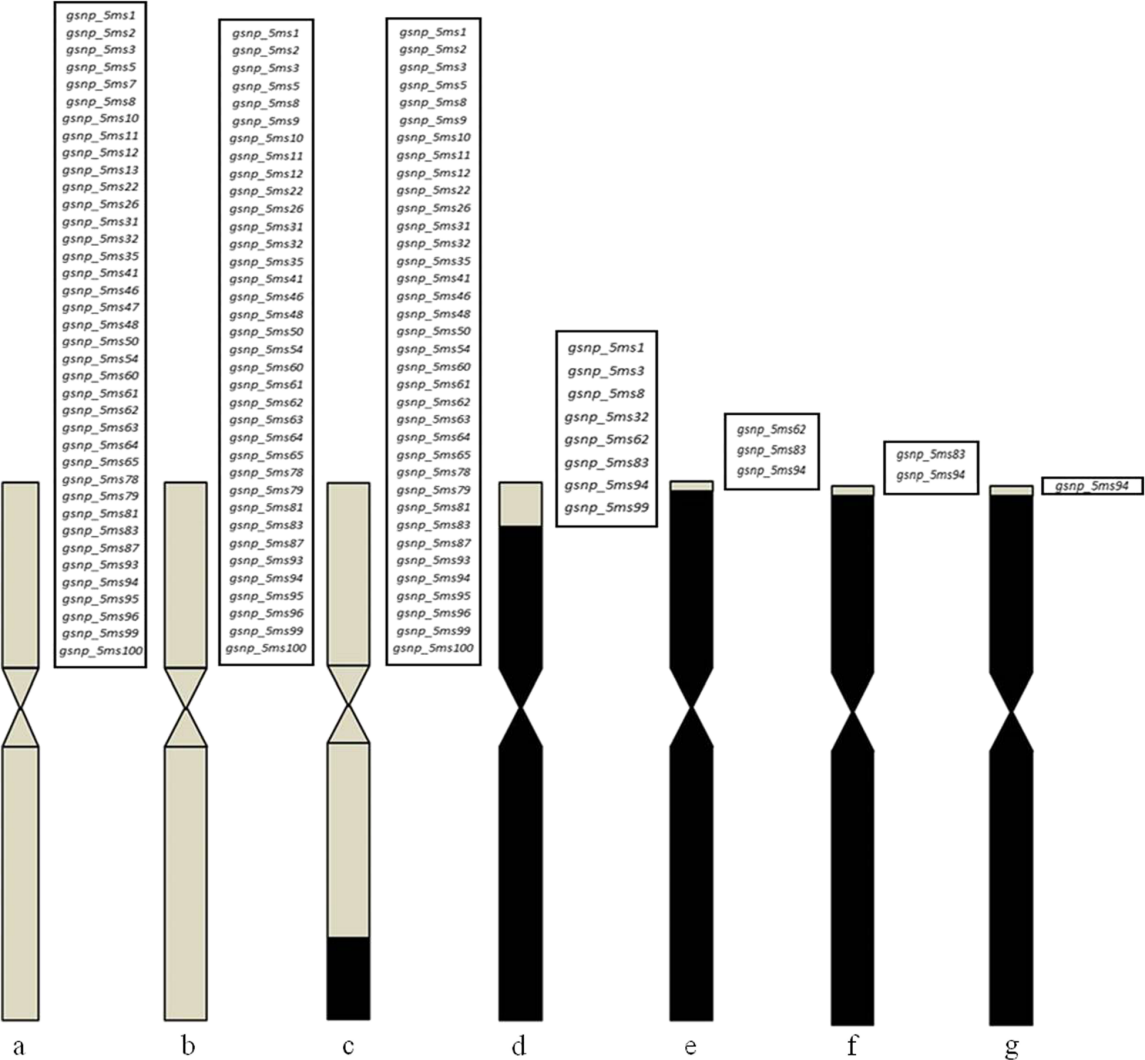
**(a-g) Distribution of validated 5M**^**g**^**S-specific SNPs developed from flow-sorted ditelosomic *5M***^***g ***^**in different alien introgression based addition, translocation and released germplasm lines. (a)** disomic addition line TA7657, **(b)** disomic substitution line TA6675, **(c)** translocation line TA5599, **(d)** terminal translocation line TA5602, **(e)** TA5602 (with very small 5M^g^ segment), **(f)** SNPs validated in germplasm KS11WGGRC53-J and **(g)** SNP validated in germplasm KS11WGGRC53-O.

Out of 44 SNPs tested on *T. aestivum* cv. WL77 and *Ae. geniculata* TA10437, 36 SNPs were validated and showed heterozygous alleles in alien translocation lines TA6675 and TA5599 (Figure 3b and c). Nine of 36 SNPs (25%) had a heterozygous allele in TA5601 (Figure 3d), and three SNPs (8.3%) were present (Figure 3e) in the translocation line TA5602.

Two germplasm lines (KS11WGGRC53-J and KS11WGGRC53-O) were developed using TA5602. Of the three SNPs (*gsnp_5ms62, gsnp_5ms83* and *gsnp_5ms94*) validated in terminal translocation line TA5602 (Figure 3e), two (*gsnp_5ms83* and *gsnp_5ms94*) mapped in KS11WGGRC53-J (Figure 3f). Only one SNP (*gsnp_5ms94*) mapped in germplasm line KS11WGGRC53-O (Figure 3g).

## Discussion

The gene pools with the wild relatives of crops hold tremendous potential for crop improvement for resistance to biotic and abiotic stresses. In wheat, many alien introgression and translocation lines involving members from tertiary gene pool have been produced [20]. Exploiting alien germplasm has been slow because of the time-consuming process of interspecific hybridization and isolation of addition and translocation lines with reduced linkage drag [19,20]. A number of approaches are available to minimize alien chromatin for reducing linkage drag [20,21,45-49]; the most widely used approach is to induce meiotic recombination between alien chromosome and its homoeologous (partially homologous) wheat chromosome. However, the frequency of homoeologous recombination between wheat and alien chromosomes is low (~3%), and a large number of progeny must be screened to isolate recombinants with small alien transfers [50]. Cytological methods, such as chromosome banding, genomic in situ hybridization (GISH) and fluorescent in situ hybridization (FISH), have been used extensively to identify introgression and translocation lines in wheat [22,51-53], but these approaches are low throughput and have resolution limitations [25,43]. Molecular markers have been used to identify alien fragments; but limited availability of alien, chromosome-specific molecular markers, especially for the tertiary gene pool species, hampers the characterization of useful lines with traits of interest [50]. As an example, only a small number of U- and M-genome-specific SSR markers are available, greatly limiting marker-assisted selection of wheat-*Ae. geniculata* and *wheat-Ae. biuncialis* introgression lines [54-56]. Practically no non-radioactive markers were available for the analysis of the wheat-*Ae. geniculata* introgressions described herein, and all previous mapping was done using RFLP analysis [43]. Qi et al. [9] used EST-STS markers to identify alien introgression lines, but polymorphism was very low and only three or fewer polymorphic markers were discovered for each arm [9]. Because the selection was based on only a few markers, useful smaller translocation may have escaped detection. Therefore, the development of a system for a user-friendly high-throughput method of identifying alien chromosome(s)-based introgression and translocation lines as reported here is highly significant.

Flow cytometric chromosome sorting has been a foundation of the genomics of the *Triticeae* tribe [57-60]. Molnár et al. [34] applied the flow-sorting technique on the allotetraploid species *Ae. biuncialis* and *Ae. geniculata* and their diploid progenitors *Ae. umbellulata* and *Ae. comosa *[34,35]. Their results provided an opportunity for the molecular analysis of wild *Aegilops* chromosomes and developing *Aegilops* chromosome-specific markers. To date, only a few studies have reported on the development of U- and M-genome-specific SSR markers [34,35,50]. In this study, we flow-sorted a ditelosomic addition wheat-*Ae. geniculata* line to isolate the short arm of the *Ae. geniculata* 5M^g^ chromosome with ~95% purity. This approach reduced DNA sample complexity and permitted the development of markers specific for the short arm of 5M^g^ (Figure 1). Approximately 145 million reads (~95%) of total sequence were obtained for mapping after trimming the data (~45X), and the deep sequencing data was used for SNP discovery.

SNP discovery using next generation sequencing (NGS) was successfully used in small-genome plants, such as *Arabidopsis* and rice, because their reference genomes are available [61,62]. SNP discovery in complex genomes without a reference genome, such as wheat [28,63] and barley [64,65], can be achieved through NGS. However, the lack of accurate reference genome sequences can create ambiguities in SNP calling, which can be further complicated by the presence of paralogs and DNA repeats [66]. We used stringent mapping parameters to minimize erroneous base calling and misaligned reads. Our SNP discovery was greatly assisted by the availability of 5AS, 5BS and 5DS chromosome shotgun sequences and assemblies developed under the International Wheat Genome Sequencing Consortium Survey Sequencing Initiative. Only about 30%, 23% and 25% of 5M^g^ short arm reads could be mapped to the 5AS, 5BS and 5DS assemblies, respectively, due to the low coverage of 5M^g^S sequences on reference assemblies, small reads and probably the diversity of the sequences. Our SNP-filtering criterion was similar to that in previously published reports [67,68] and yielded 277,770 (5AS), 203,522 (5BS) and 355,765 (5DS) high-quality SNPs (Table 1). In this research we generated single end sequencing reads, assembled data provided us ~7000 contigs > =500 bp. Blast against wheat EST database of these larger contigs suggested ~1400 contigs with genes on *Ae. geniculata* chromosome arm 5M^g^S.

The estimated SNP frequency in our study was ~1.3 SNPs/kb of the total analyzed sequences. This frequency is slightly lower than those of previous reports; Trick et al. [67] found an average density of 1.80 (±1.46) SNP/kb and Ravel et al. [68] estimated SNP frequency to be 2.99 SNP/kb [68]. The lower estimated SNP frequency can be attributed to the low coverage of 5M^g^ sequences on reference assemblies and a stringent filtering criterion. For developing M-genome-specific SNPs, we needed identical SNP alleles in 5AS, 5BS and 5DS but different in 5M^g^S. We aligned the 100-bp flanking sequences of the SNPs that were discovered based on the three references (5AS, 5BS and 5DS contigs) and only those sequences were selected that showed 100% similarity on the flanking sequences of the SNP. Critical selection of SNPs against 5AS, 5BS and 5DS yielded 2,178 reliable 5M^g^-specific SNPs which were around 1% of total SNPs discovered.

Our study identified ~2,178 chromosome 5M^g^S-specific SNPs, providing a quick approach for developing markers that would facilitate identifying alien addition and translocation lines. For M-genome-specific marker development, we shortlisted 104 sequences with unique SNPs. Using two different SNP validation approaches, we tested 59 randomly selected SNPs in wheat cultivar Chinese Spring and *Ae. geniculata* TA2899, and the validation rate was found to be ~84%. We tested Chinese Spring, WL711, and seven *Ae. geniculata* accessions. When comparing Chinese Spring against all the *Ae. geniculata* accessions, the average validation was 78.4%, with a range of 68.8% to 85.4%. For another set involving wheat line WL711 and the seven *Ae. geniculata* lines, the average validation rate was 76.0%, with a range of 65.7% to 81.4%. These results indicate the fixation of more than 70% of 5M^g^-specific alleles in *Ae. geniculata* accessions (TA1800, TA1801, TA2847, TA2899, TA10029, TA2041 and TA10437), suggesting the usefulness of these SNPs in multiple *Ae. geniculata* accessions. We compared the validation rates of 59 SNPs (33 genic and 26 non-genic sequences). The validation rate for the genic and non-genic SNPs was very similar (86.1% and 85.2%, respectively). The validated 5M^g^S-specific markers (Additional file 3: Table S1) will be useful for monitoring introgression (5M^g^S) in *Ae. comosa*, *Ae. geniculata* and *Ae. biuncialis,* because they share a common M genome*.* The KASPar assay used in this study provides a cheap and high-throughput means for identifying alien introgressions because one assay mix is sufficient for ~2,500 reactions. Validated SNPs between Chinese Spring and *Ae. geniculata* TA2899 were used to identify an alien disomic addition line (TA7659). Approximately 97.3% of the validated SNPs identified the addition line with heterozygous alleles (Figure 2b), confirming the presence of both wheat and *Ae. geniculata* chromosomes in TA7659. Forty-four 5M^g^S-specific SNPs also were tested on WL711, the *Ae. geniculata* accession TA10437 and a set of their substitution and translocation lines. As expected 94.4%, 94.4%, 25%, and 8.3% of the validated SNPs showed heterozygous alleles in TA6675, TA5599, TA5601 short arm and TA5602, respectively. These data clearly indicate the applicability of SNP-based identification for alien addition, substitution and translocation lines.

We also validated two released germplasm lines (KW11wggrc53-J and KS11wggrc53-O) developed by crossing TA5602 with two susceptible winter wheat lines Jagger and Overley. Both lines were found to have 5M^g^S-specific SNPs mapped on TA5602 suggesting that two markers (*gsnp_5ms83* and *gsnp_5ms94*) could be used for marker-assisted selection for disease resistance genes (*Lr57* and *Yr40*).

## Conclusions

**Figure 4:**
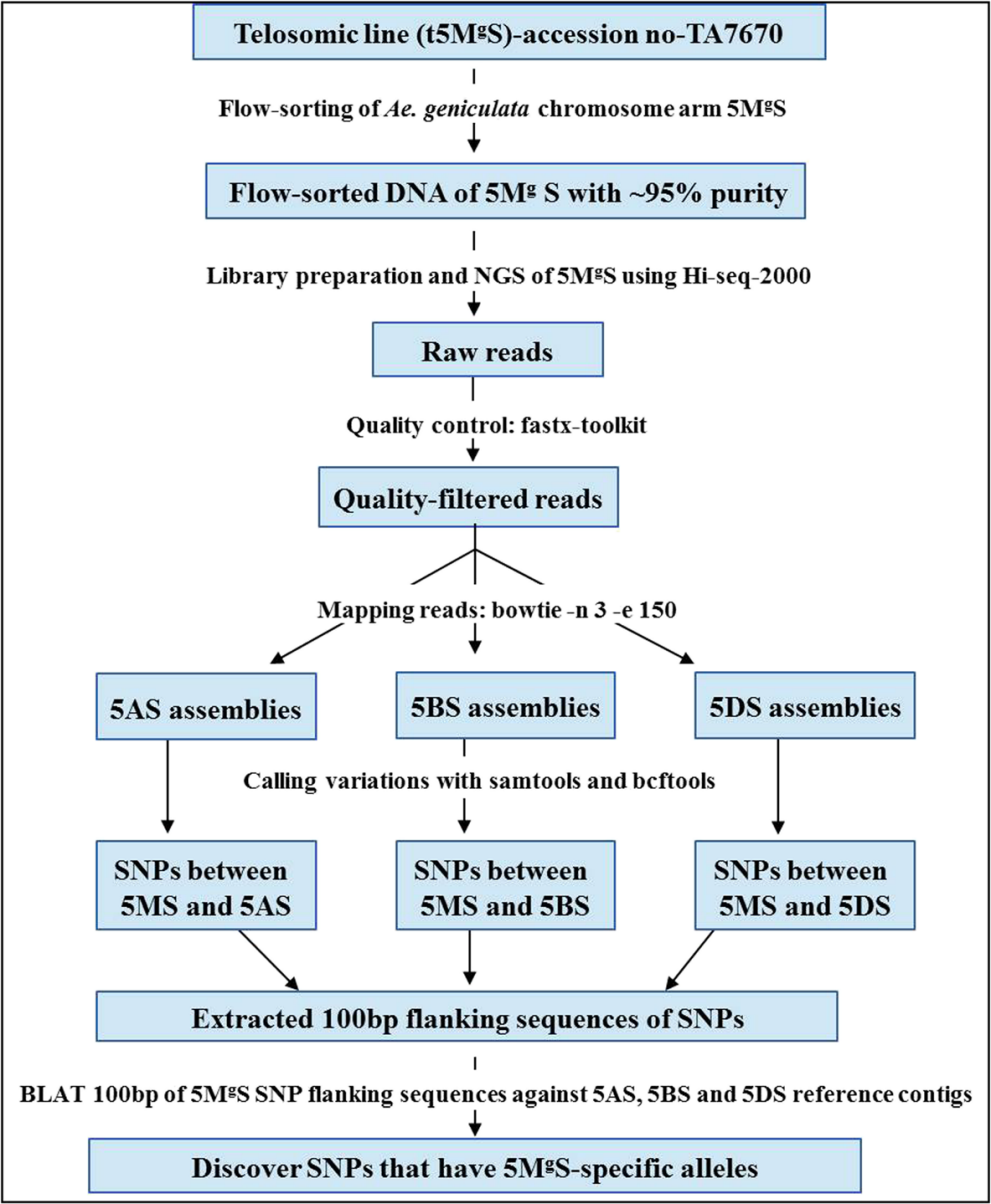
**Graphical summary scheme.** A schematic showing the strategy used for genome specific SNP discovery for 5M^g^S of *Ae. geniculata*.

To conclude, this study marks an important step forward for utilizing wild and related resources of wheat. For the first time, an arm of an *Aegilops* chromosome from the tertiary gene pool of wheat was successfully flow-sorted and sequenced by Illumina technology. Recently it has been established that using ‘Fluorescence in situ hybridization in suspension’ approach (FISHIS) individual *Aegilops* chromosomes can be flow-sorted with high purity from wheat-*Aegilops* disomic addition and substitution lines (Dolezel et al. personal communication). Alternatively, advances in flow cytogenetics have made possible even the flow-sorting of *Aegilops* chromosomes from respective *Aegilops* species [35]. The approach reported in this article can be used for marker development from targeted flow-sorted *Aegilops* chromosome(s) and their applications in marker assisted selection (Figure 4). Next-generation sequencing offers a cheap way to develop sequence-based markers for molecular analysis of *Aegilops* chromosomes. The ability to purify chromosome arms of *Aegilops* species will be very useful for physical mapping, constructing arm-specific BAC libraries and developing NGS-based genic and low-copy sequences to make chromosome- and genome-specific markers. Recently released flow-sorted chromosome arm based survey sequenced assemblies of all wheat chromosomes will be very useful in alien chromosome based genome specific SNP discovery (https://urgi.versailles.inra.fr/download/iwgsc/). Mapped arm- and genome-specific SNP markers can be used to identify alien chromosome segments with a gene of interest for pre-breeding in wheat improvement programs.

## Methods

### Plant material

All plant materials used in this study were procured, developed and maintained by the Wheat Genetics Resource Center at Kansas State University (http://www.k-state.edu/wgrc/). Plant materials include *Ae. geniculata* (accession TA2899) 5M^g^ chromosome disomic addition line (TA7659) in wheat cultivar Chinese Spring [42], a disomic substitution line [TA6675; DS5M^g^(5D)], and three wheat-*Ae. geniculata* translocation lines TA5599, TA5601, and TA5602 with 75%, 25%, and 5% of *Ae. geniculata* (accession TA10437) 5M^g^ chromosome, respectively [43,49]. Disomic addition line TA7659 was used to develop a ditelosomic (t5M^g^S) line (TA7670) in wheat [42]. Translocation lines TA5599 [T5DL-5M^g^L · M^g^S (0.25)] and TA5601 [T5DL · 5DS-5M^g^S (0.75)] are BC_2_F_5_ hybrids derived from a disomic substitution line TA6675 [DS5M^g^(5D)] and Chinese Spring (CS)*Ph*^*I*^ stock [48], followed by backcrossing to the bread wheat cultivar WL711 [43,49]. Translocation line TA5602 [T5DL · 5DS-5MgS (0.95)] is a BC_3_F_6_ line derived in a similar way [43]. All the three translocation lines (TA5599, TA5601 and TA5602) carry *Lr57* and *Yr40* genes. A set of *Ae. geniculata* accessions (TA2899, TA1800, TA1801, TA2049, TA2786, TA10437 and TA10029) and wheat lines (Chinese Spring, WL711, Jagger and Overley) were also used in this study. Two germplasm lines (KS11WGGRC53-J and KS11WGGRC53-O) released by the WGRC (http://www.k-state.edu/wgrc/Germplasm/grmplsm.html) were used to validate the 5M^g^S SNPs mapped in TA5602. The germplasm KS11WGGRC53-J was developed by crossing the translocation line TA5602 and winter wheat cultivar Jagger (TA5602/3*Jagger; TA5089-L1) whereas KS11WGGRC53-O was developed by crossing TA5602 with winter wheat cultivar Overley (TA5602/3*Overley; TA5089-L2).

### Flow-sorting and next-generation sequencing

Aqueous suspensions of mitotic metaphase chromosomes were prepared from wheat-*Ae. geniculata* ditelosomic addition line 5M^g^S (TA7670) [42] following the protocol of Vrána et al. [58]. The samples were stained by DAPI and analyzed using an FACSAria II SORP flow cytometer and sorter (BD Biosciences, San Jose, USA). Three independent samples of 100,000 chromosomes were sorted into 40 μl of sterile deionized water in a 0.5-ml PCR tube. The contamination of sorted fractions by other chromosomes was determined for each sorted run after analyzing 1,000 chromosomes on a microscopic slide. The chromosomes were identified using FISH with probes for the Afa-family and [GAA]n repeats [59]. The DNA of chromosomes sorted into PCR tubes was purified and amplified using an Illustra GenomiPhi V2 DNA Amplification Kit (GE Healthcare, Piscataway, USA) as described by Šimková et al. [69]. The three samples of amplified DNA were pooled to reduce possible amplification bias. Sequencing of amplified chromosomal DNA was performed with HiSEq 2000 (Illumina). Pooled MDA-amplified DNA was used to create the corresponding shotgun DNA-seq library. The library for the 5M^g^S was run in a single lane at DNA core facility services at University of Missouri, USA. For this project we opted for single read sequencing where only one end is sequenced. Sequence data generated from the short arm of *Ae. geniculata* chromosome arm 5M^*g*^ was used for SNP discovery.

### Rationale and strategy for read alignment and variant calling

Before aligning reads to references, all reads were first trimmed to remove the low-quality bases (phred score < =15) on the end of the reads; reads with more than 30 bp after quality trimming were then filtered with an overall quality (80% of bases must have quality ≥15). NGS-based SNP discovery involved two basic steps: (1) alignment of NGS on a reference genome sequence, also called read mapping, and (2) variant calling from the aligned sequences. Variant calling is much easier if a reference sequence is available, because short reads with deep sequencing coverage increases the confidence level for SNP discovery. In this study, we used Chromosome Shotgun Sequences (CSS) as reference sequences and assemblies from wheat chromosome arms 5AS, 5BS and 5DS developed under the International Wheat Genome Sequencing Consortium Survey Sequencing Initiative (http://www.wheatgenome.org/). The 5M^g^S reads were mapped on these assemblies. In the second step, variants were called against each individual assembly (5AS, 5BS and 5DS). We used the Bowtie software (v1.3) (http://bowtie-bio.sourceforge.net/index.shtml) to map quality filtered reads of 5M^g^ to the Chinese Spring 5A, 5B and 5D contigs separately (Figure 4). The parameters to control the mapping quality were: -k 1 –best –v 3. The alignment results were saved in SAM format files. SAMtools (v1.8) (http://samtools.sourceforge.net/) were used to generate pileup files, which were then fed to BCFtools (http://samtools.sourceforge.net/) to call raw variations using default parameters. All variations with coverage depth > =4, SNP quality > =50 were kept for subsequent analysis (Figure 4). We also assembled 5M^g^S contigs using the SOAPdenovo (http://soap.genomics.org.cn/soapdenovo.html) to assemble contigs for the 5M^g^ genome, with parameter -k 27.

### Detection of M^g^-genome-specific SNPs

To identify 5M^g^-specific SNPs, the program BLAT was used to align the 100-bp flanking sequences of 5M^g^S-specific SNPs against the 5AS, 5BS and 5DS reference contigs. The alleles of SNPs that had 100% similarity on the flanking sequences in 5AS, 5BS and 5DS were then compared to select the potential 5M^g^S-specific SNPs.

### Validation of SNPs

#### *KASPar genotyping based validation*

A set of 44 SNPs were selected for further validation and identification of alien addition and translocation lines. For each putative 5M^g^S-specific SNP, two allele-specific forward primers and one common reverse primer (Additional file 3: Table S1) were designed (KBioscience, Hoddesdon, UK). Genotyping reactions were performed in a final volume of 8.11 μl, which included a reaction mix: 4.0 μl of 2x reaction mix (per reaction) (KBioscience, Hoddesdon, UK), and 0.11 ul assay mix (per reaction) and ~20 ng of genomic DNA (4.0 μL). The following cycling conditions were used: 94°C for 15 minutes; and 35 cycles of 94°C for 10 seconds and 60°C for 1 minute, followed by 35°C for 30 seconds for plate reading. All reactions used a CFX96 Touch™ Real-Time PCR Detection System (Bio-Rad, Hercules, CA, USA), which has an inbuilt fluorescence scanner, and data were analyzed using Bio-Rad CFX manager software under the allelic discrimination mode.

#### *PCR product-based SNP validation*

To assess the veracity of the discovered SNPs and estimate the false-positive SNP discovery rate, 15 sequences, with one SNP each, were randomly chosen from the SNP reference sequences. SNP flanking primers were designed with Primer3 (http://bioinfo.ut.ee/primer3-0.4.0/). Of 15 primers, only 12 primer pairs generated PCR products in both Chinese Spring and *Ae. geniculata* TA2899. Failure to amplify the target DNA by two primer pairs was due to suboptimal PCR conditions, later confirmed by optimization. The PCR products were eluted from the electrophoresis gels and purified. Purified products were then sequenced with an Applied Biosystems 3730xl DNA Analyzer (Life Technologies, USA) and sequences were aligned and compared for SNPs.

### Availability of supporting data

The 5M^g^ short arm sequence data has been submitted to SRA under accession SRX474187. An additional table and two additional figures were also included in the manuscript.

## Competing interests

The authors declare that they do not have any competing interests.

## Authors’ contributions

All the listed authors contributed significantly to the manuscript. VKT, JS, SKS and BSG planned the research. BF, BK and PC contributed to the germplasm development used in the experiments. JV, MK and JD carried out chromosome flow-sorting and DNA amplification experiments. VKT, SW, SKS and EA contributed to DNA sequencing, sequence analysis, SNP discovery, validation and introgression mapping experiments. VKT and BSG prepared the manuscript. All authors read and approved the final manuscript.

## Supplementary Material

Additional file 1: Figure S1 The frequency and coverage depth of 5M^g^S assembled sequences. Click here for file

Additional file 2: Figure S2 Coverage depth of 5M^g^S sequences against reference contigs 5AS, 5BS and 5DS. Click here for file

Additional file 3: Table S1 List of 104 5M^g^S-based, genome-specific SNP markers developed by mapping the 5M^g^ Illumina reads on 5AS, 5BS and 5DS assemblies of *T. aestivum* cv. Chinese Spring. Click here for file
