# Supplementary figures and images for: SNP Discovery for mapping alien introgressions in wheat
Source: BMC Genomics. 2014 Apr 10;15:273. doi: 10.1186/1471-2164-15-273 (PMC4051138; doi:10.1186/1471-2164-15-273)

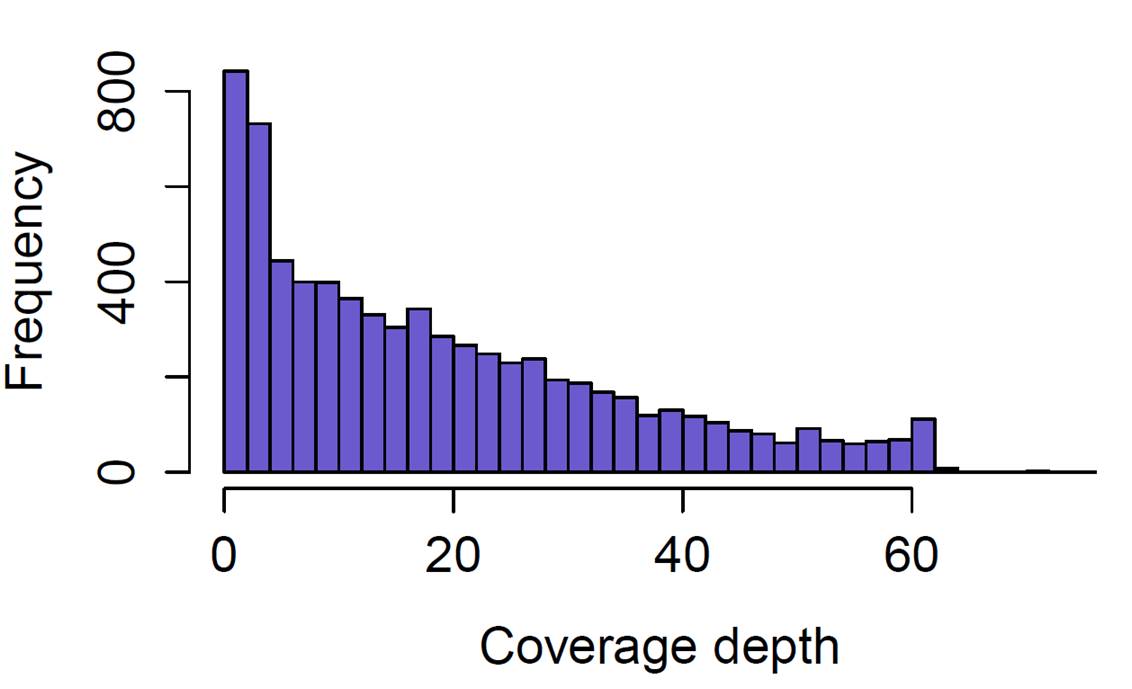

Supplement: Additional file 1: Figure S1 — The frequency and coverage depth of 5MgS assembled sequences. [file 1471-2164-15-273-S1.JPEG]

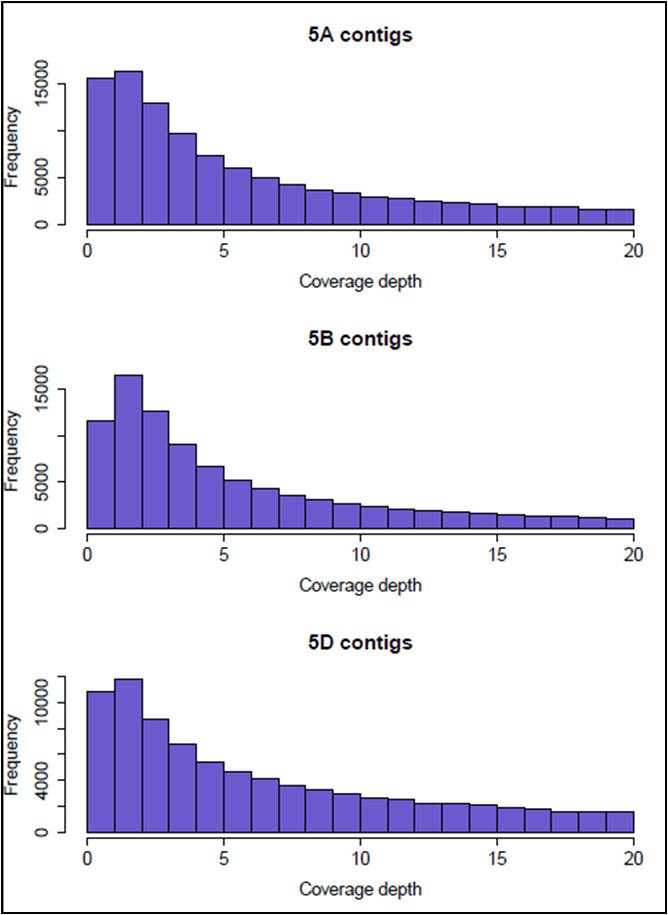

Supplement: Additional file 2: Figure S2 — Coverage depth of 5MgS sequences against reference contigs 5AS, 5BS and 5DS. [file 1471-2164-15-273-S2.JPEG]
